# Calcium dysregulation contributes to neurodegeneration in FTLD patient iPSC-derived neurons

**DOI:** 10.1038/srep34904

**Published:** 2016-10-10

**Authors:** Keiko Imamura, Naruhiko Sahara, Nicholas M. Kanaan, Kayoko Tsukita, Takayuki Kondo, Yumiko Kutoku, Yutaka Ohsawa, Yoshihide Sunada, Koichi Kawakami, Akitsu Hotta, Satoshi Yawata, Dai Watanabe, Masato Hasegawa, John Q. Trojanowski, Virginia M.-Y. Lee, Tetsuya Suhara, Makoto Higuchi, Haruhisa Inoue

**Affiliations:** 1 Center for iPS Cell Research and Application (CiRA), Kyoto University, Kyoto, Japan; 2 Department of Functional Brain Imaging Research, National Institute of Radiological Sciences, National Institutes for Quantum and Radiological Science and Technology, Chiba, Japan; 3 Department of Translational Science and Molecular Medicine, College of Human Medicine, Michigan State University, Grand Rapids, MI, USA; 4 Department of Neurology, Kawasaki Medical School, Kurashiki, Okayama, Japan; 5 Division of Molecular and Developmental Biology, National Institute of Genetics and Department of Genetics, SOKENDAI (The Graduate University for Advanced Studies), Mishima, Shizuoka, Japan; 6 Department of Biological Sciences, Graduate School of Medicine, Kyoto University, Kyoto, Japan; 7 Department of Dementia and Higher Brain Function, Tokyo Metropolitan Institute of Medical Science, Tokyo, Japan; 8 Center for Neurodegenerative Disease Research, Institute on Aging, University of Pennsylvania Perelman School of Medicine, Philadelphia, USA

## Abstract

Mutations in the gene *MAPT* encoding tau, a microtubules-associated protein, cause a subtype of familial neurodegenerative disorder, known as frontotemporal lobar degeneration tauopathy (FTLD-Tau), which presents with dementia and is characterized by atrophy in the frontal and temporal lobes of the brain. Although induced pluripotent stem cell (iPSC) technology has facilitated the investigation of phenotypes of FTLD-Tau patient neuronal cells *in vitro*, it remains unclear how FTLD-Tau patient neurons degenerate. Here, we established neuronal models of FTLD-Tau by Neurogenin2-induced direct neuronal differentiation from FTLD-Tau patient iPSCs. We found that FTLD-Tau neurons, either with an intronic *MAPT* mutation or with an exonic mutation, developed accumulation and extracellular release of misfolded tau followed by neuronal death, which we confirmed by correction of the intronic mutation with CRISPR/Cas9. FTLD-Tau neurons showed dysregulation of the augmentation of Ca^2+^ transients evoked by electrical stimulation. Chemogenetic or pharmacological control of neuronal activity-relevant Ca^2+^ influx by the introduction of designer receptors exclusively activated by designer drugs (DREADDs) or by the treatment with glutamate receptor blockers attenuated misfolded tau accumulation and neuronal death. These data suggest that neuronal activity may regulate neurodegeneration in tauopathy. This FTLD-Tau model provides mechanistic insights into tauopathy pathogenesis and potential avenues for treatments.

Late-onset neurodegenerative diseases are becoming a growing burden to society due to the gradual increase in life expectancy and dramatic rise in prevalence of these diseases. Accumulating evidence strongly implicates misfolded proteins as a causative agent for most neurodegenerative diseases^1^,^2^,^3^,^4^,^5^ including neurodegenerative tauopathies^6^,^7^,^8^,^9^,^10^, which are diseases associated with the pathological aggregation of microtubule-associated protein tau in the brain. Mutations in tau gene (*MAPT*) cause a familial disease known as frontotemporal lobar degeneration tauopathy or FTLD-Tau, which presents with dementia and is characterized by prominent atrophy in the frontal and temporal lobes of the human brain.

Tau is axonal microtubule-associated protein that contributes to the stabilization of microtubules. There are six tau isoforms, the result of the splicing of exons 2, 3 and 10. Alternative splicing of exon 10, which encodes the microtubule-binding domains (MBDs), generates 3-repeat tau with 3 MBDs or 4-repeat tau with 4 MBDs. Exonic mutations in *MAPT* are known to attenuate the ability of tau to bind to microtubules, accelerate self-aggregation, and alter *MAPT* splicing^11^,^12^,^13^. Intronic mutations in *MAPT* are shown to affect exon 10 splicing and increase 4 repeat tau, which is accumulated in postmortem brain of patients with an intronic mutation^14^,^15^.

Although the developments in induced pluripotent stem cell (iPSC) technology have facilitated the investigation of phenotypes of neurodegenerative diseases including FTLD-Tau patient neural cells *in vitro*^16^,^17^,^18^,^19^, the mechanism involved in neurodegeneration in FTLD-Tau patient neurons is still unclear. Here, we established an FTLD-Tau iPSC model using Neurogenin2 (Ngn2)-induced direct neuronal differentiation from FTLD-Tau patients either with an intronic or with an exonic *MAPT* mutation^20^. This direct conversion method produces a robust amount of cortical neurons. We also generated an isogenic control iPSCs by the clustered regularly interspaced short palindromic repeats (CRISPR)/CRISPR-associated protein 9 (Cas9) system, and observed accumulation and extracellular release of misfolded tau protein followed by neuronal death resulting in the recapitulation of the converged phenotypes of *MAPT* intronic and exonic mutations. Furthermore, to explore the mechanism of neurodegeneration in FTLD-Tau, we generated FTLD-Tau iPSCs constitutively expressing designer receptors exclusively activated by designer drugs (DREADDs), and found that calcium dysregulation contributed to the neurodegeneration.

## Results

**Figure 1:**
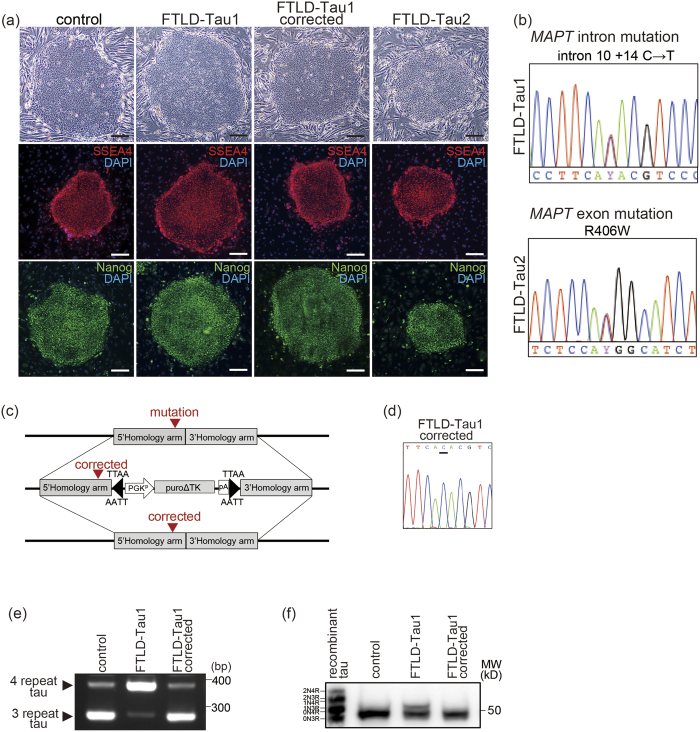
Generation of iPSCs from FTLD-Tau patients with either intronic or exonic *MAPT* mutation and gene editing for isogenic control. (**a**) iPSCs generated from familial FTLD-Tau patients with a *MAPT* mutation showed ESCs-like morphology (phase image) and expressed the pluripotent stem cell markers SSEA4 and Nanog. Scale bar = 100 μm. (**b**) The FTLD-Tau1 iPSC line carried a *MAPT* intron mutation (intron 10 + 14C → T), and the FTLD-Tau2 iPSC line carried a *MAPT* exon mutation (R406W). (**c,d**) The mutation in FTLD-Tau1 was corrected (FTLD-Tau1 corrected) using the CRISPR/Cas9 system. Sanger sequence analysis revealed correction of the mutation after gene editing. (**e**) RT-PCR showing FTLD-Tau1 neurons with increased 4 repeat tau expression, and FTLD-Tau1 corrected neurons with the normal expression ratio of 4 repeat and 3 repeat tau. Full-length picture of the gel is presented in Figure S4(a). (**f**) Cell lysates were dephosphorylated with lambda phosphatase and separated by SDS-PAGE for comparison with recombinant tau ladder followed by detection using an anti-total tau antibody (Tau12). Altered band pattern of FTLD-Tau1 was reversed in FTLD-Tau1 corrected. Full-length picture of the blot is presented in Figure S4(b).

**Figure 2:**
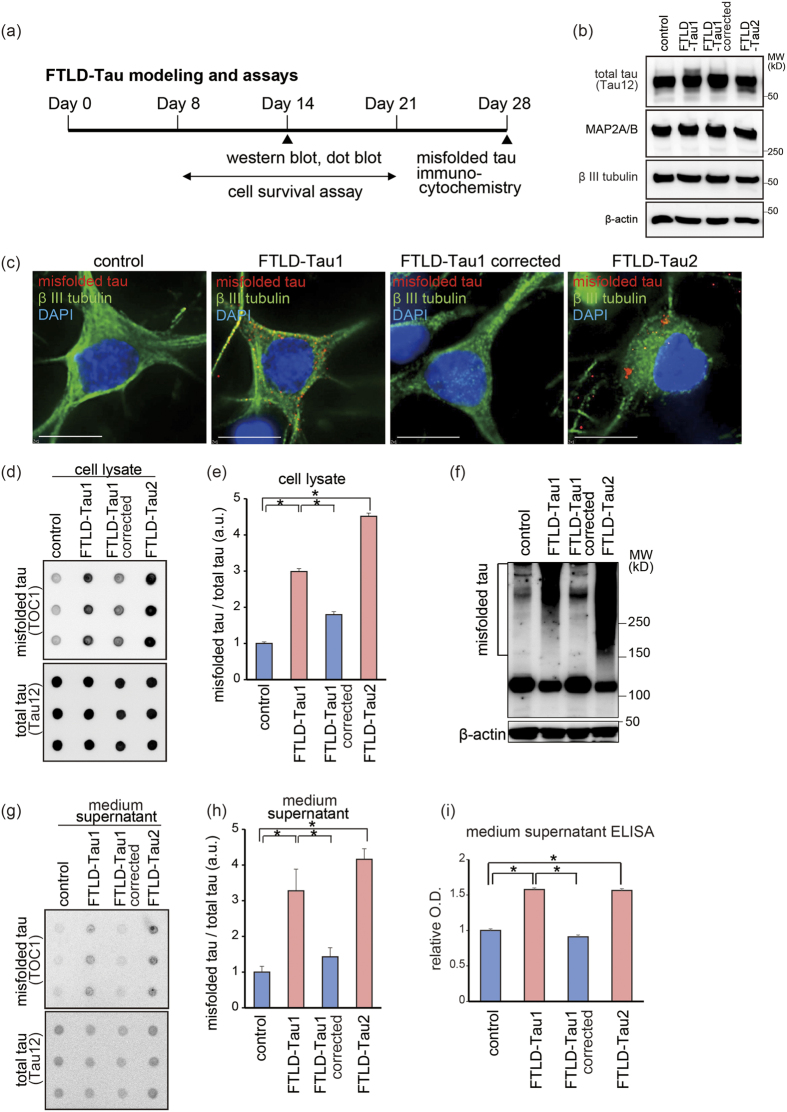
Accumulation of misfolded tau in FTLD-Tau neurons. (**a**) An experimental time line for FTLD-Tau modeling. (**b**) Western blot analysis showing total tau, MAP2A/B, and βIII tubulin in generated neurons. There were no significant differences in MAP2A/B levels among the neuronal lines, indicating no significant difference of neuronal differentiation. Full-length pictures of the blots are presented in Figure S4(c–f). (**c**) Intracellular misfolded tau accumulation in FTLD-Tau neurons (green, βIII tubulin-positive cells) was detected by TOC1 antibody (red) with a punctate pattern. Scale bar = 10 μm. (**d,e**) Dot blot analysis of FTLD-Tau neurons. The ratios of misfolded tau/total tau were increased in cell lysates of FTLD-Tau neurons (n = 3; one way ANOVA, p < 0.05; *post hoc* test, *p < 0.05). (**f**) Western blot analysis using TOC1 antibody under non-reducing condition showing misfolded high molecular tau in cell lysates of FTLD-Tau neurons. Full-length pictures of the blots are presented in Figure S4(g,h). (**g,h**) Dot blot analysis of FTLD-Tau neurons. The ratios of misfolded tau/total tau were increased in culture medium of FTLD-Tau neurons (n = 3; one way ANOVA, p < 0.05; *post hoc* test, *p < 0.05). (**i**) Misfolded tau in the culture medium supernatant was quantified by ELISA using TOC1 antibody for capture and anti-human tau antibody for detection. The level of misfolded tau was increased in culture medium of FTLD-Tau neurons (n = 3; one-way ANOVA, p < 0.05; *post hoc* test, *p < 0.05).

We generated iPSCs from FTLD-Tau patients with a mutation, either intron 10 + 14C → T^14^, or exon R406W^21^ (Figs 1(a,b) and S1(a,b) and Table S1). These patients presented frontotemporal dementia. The intron 10 + 14C → T mutation was corrected using CRISPR/Cas9 (Fig. 1(c,d)). Ngn2 was introduced into these iPSCs via a *piggyBac* vector with a tet-on expression system, followed by drug selection for stable-line establishment. The established iPSCs were converted to cortical neurons after 7 days of culture in neuronal medium with doxycycline. There were no differences in the differentiation propensities of the resulting lines; the percentages of neurons in control, FTLD-Tau1, FTLD-Tau1 corrected, and FTLD-Tau2 lines were 89.5 ± 1.9%, 91.2 ± 0.9%, 90.6 ± 1.6%, and 87.6 ± 0.2%, respectively (n = 3). The generated neurons expressed mRNA of receptors of neurotransmitters (Figure S1(c)), and electrophysiological analysis presented their functional properties (Figure S2(a–h)). FTLD-Tau1 neurons with the intron 10 mutation showed increased 4-repeat tau expression compared with the 3-repeat tau expression, as previously reported^14^, and correction of the mutation repaired the ratio (Fig. 1(e,f)). We modeled FTLD-Tau using these FTLD-Tau and control neurons (Fig. 2(a)). The differentiated neurons exhibited neuron marker MAP2A/B, and these levels were not different between the respective lines (Fig. 2(b)). FTLD-Tau neurons, both with the intronic *MAPT* mutation and with the exonic mutation harbored accumulations of intracellular misfolded tau detected by immunocytochemistry using an anti-oligomeric aggregate antibody, TOC1 antibody^22^,^23^ (Figs 2(c) and S3(a) and Table S2). Some FTLD-Tau neurons exhibited typical misfolded tau puncta and dots, and control neurons including the gene-corrected line were mostly negative for misfolded tau puncta or dots. Dot blot analysis presented accumulation of intracellular misfolded form of tau in non-denaturing condition using TOC1 antibody (Fig. 2(d,e)). We also analyzed misfolded tau by western blot analysis using TOC1 antibody to detect tau species in a denaturing condition as shown previously^23^. FTLD-Tau neurons either with the intronic *MAPT* mutation or the exonic mutation exhibited accumulations of tau species with higher molecular weight than control (Fig. 2(f)). However, there was a difference in molecular weight shifting of misfolded tau between FTLD-Tau neurons with the intronic *MAPT* mutation and with the exonic mutation (Fig. 2(f)), suggesting that conformation of misfolded tau might not be uniform in the denaturing condition. Furthermore, FTLD-Tau neurons presented misfolded tau release into culture medium as detected by dot blot analysis (Fig. 2(g,h)) and confirmed by ELISA (Fig. 2(i)).

**Figure 3:**
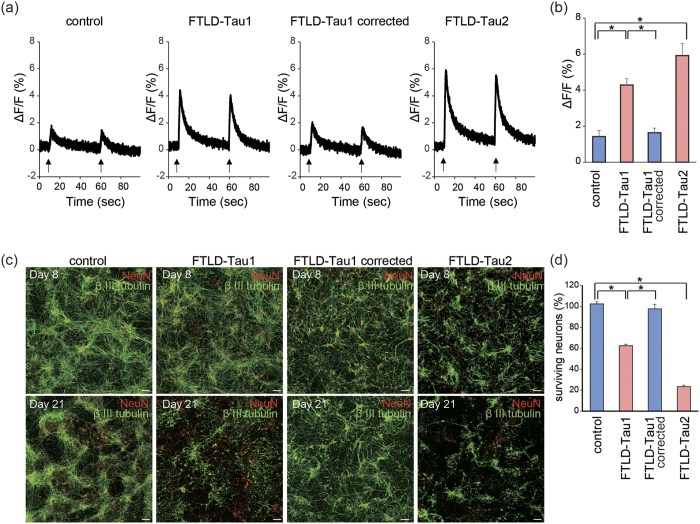
Calcium dysregulation and neuronal loss of FTLD-Tau neurons. (**a,b**) Calcium imaging after electrical stimulation. Intracellular Ca^2+^ levels, elevated by electrical stimulation, were higher in FTLD-Tau neurons than in control (n = 6; one-way ANOVA, p < 0.05; *post hoc* test, *p < 0.05). (**c,d**) Immunofluorescence staining of βIII tubulin and NeuN on Days 8 and 21 in control and FTLD-Tau neurons. Survival of FTLD-Tau neurons from Day 8 to Day 21 was decreased compared to control neurons (n = 6; one-way ANOVA, p < 0.05; *post hoc* test, *p < 0.05).

Neurons of FTLD-Tau model mice overexpressing human mutant tau^24^ and FTLD-Tau patients^25^ can show increased excitability. Furthermore, misfolded tau-exposed neurons exhibited increased intracellular Ca^2+^ levels^26^. Thus, we evaluated Ca^2+^ transient of FTLD-Tau neurons by calcium imaging assay. Electrical stimulations increased intracellular Ca^2+^ levels, and the increased ratio was greater in FTLD-Tau neurons compared with control neurons, indicating increased excitability of FTLD-Tau neurons (Fig. 3(a,b)). Next, we investigated the vulnerability of FTLD-Tau neurons to degeneration, and found that they were more vulnerable to undergoing cell death than control neurons (Figs 3(c,d) and S3(b,c)).

**Figure 4:**
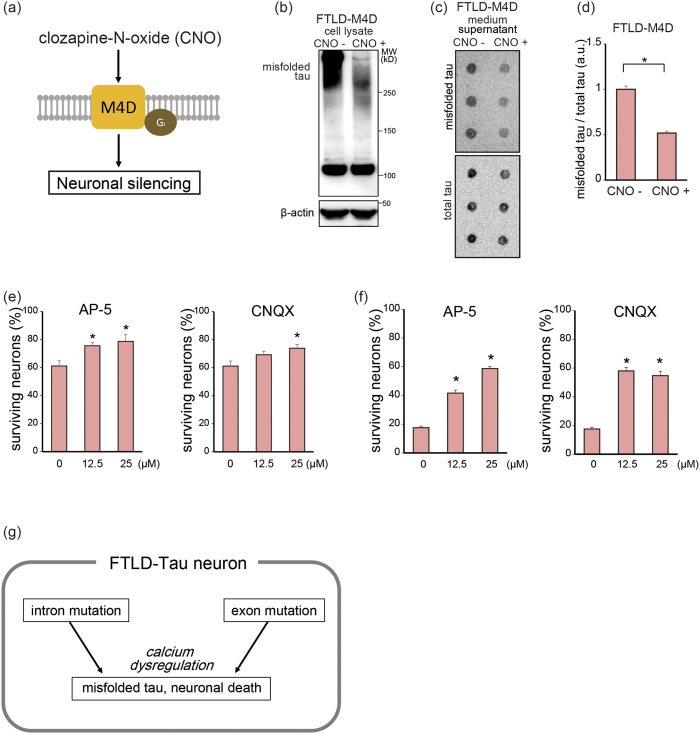
Attenuation of neurodegeneration by regulation of Ca^2+^ influx. (**a**) Schema of Designer Receptor Exclusively Activated by Designer Drug (DREADD) systems for regulating neuronal activity. (**b**) Western blot analysis of misfolded tau in FTLD-Tau neurons after SDS-PAGE under non-reducing conditions using TOC1 antibody. Stimulation of M4D neurons with CNO decreased the level of misfolded tau in cell lysates. Full-length pictures of the blots are presented in Figure S4(i,j). (**c,d**) Dot blot analysis of misfolded tau (TOC1) and total tau (Tau12) in FTLD-Tau neuron culture medium. Stimulation of M4D neurons with CNO decreased the level of extracellular misfolded tau (n = 3; Student-*t* test, *p < 0.05). (**e**) Treatment with AP-5 or CNQX increased survival of FTLD-Tau1 neurons. (n = 6; one-way ANOVA, p < 0.05; *post hoc* test, *p < 0.05). Bar graphs represent mean ± SEM. (**f**) Treatment with AP-5 or CNQX increased survival of FTLD-Tau2 neurons. (n = 6; one-way ANOVA, p < 0.05; *post hoc* test, *p < 0.05). Bar graphs represent mean ± SEM. (**g**) Summary of our findings.

We assessed the mechanistic links between the excitation of neurons and misfolded tau using DREADDs, which enabled us to control neuronal activity. We incorporated DREADDs, consisting of human muscarinic acetylcholine M4 receptor (M4D)^27^ bearing artificial mutations, into FTLD-Tau iPSCs using tol2 vectors following drug selection, and generated neurons constitutively expressing M4D (Fig. 4(a)). Stimulation of M4D by its exclusive agonist, clozapine-N-oxide (CNO), which inhibits calcium influx via Gi signaling, decreased intracellular and extracellular misfolded tau (Fig. 4(b–d)), suggesting that neuronal activity may modulate tau pathology. Furthermore, inhibition of Ca^2+^ influx by the N-methyl-D-aspartate (NMDA) receptor inhibitor D-2-amino-5-phosphonopentanoate (AP-5) and the α-amino-3-hydroxy-5-methyl-4-isoxazolepropionic acid (AMPA) receptor inhibitor 6-cyano-7-nitroquinoxaline-2,3-dione (CNQX) increased survival of FTLD-Tau neurons (Fig. 4(e,f)).

Collectively, the cellular phenotypes of FTLD-Tau neurons, including accumulation of misfolded tau and neuronal death, are similar for both the intronic and exonic *MAPT* mutations, and neuronal activity-relevant Ca^2+^ influx modulates neurodegeneration in tauopathy (Fig. 4(g)).

## Discussion

In the current study, we modeled FTLD-Tau using patients’ cortical neurons that exhibit an accumulation and extracellular release of misfolded tau and neuronal death, key findings of neurodegeneration due to tauopathy. These phenotypes were modulated by chemogenetic control of the introduced DREADDs or by glutamate receptor blockers. Our findings suggest that neurodegeneration in FTLD-Tau involves misfolded tau and it is modulated by neuronal activity.

We used iPSCs from two FTLD-Tau patients either with intronic mutation 10 + 14C → T or with exonic mutation R406W. R406W mutation is reported to reduce the binding of tau to microtubules and promote formation of misfolded tau in model mice^28^. Mutation of intron 10 is known to affect exon 10 splicing and increase the ratio of 4R tau/3R tau. Imbalances in 4R and 3R tau are believed to cause neurodegeneration, but the pathogenic mechanisms were not well known, although model mice with overexpression of the intronic mutation showed neurodegeneration^29^. We found accumulation of misfolded tau in FTLD-Tau neurons with an intron mutation to be the same as with exon mutation. Since self-aggregation of 4R tau^30^,^31^ may be associated with the accumulation of misfolded tau in the intronic mutation, the accumulation of misfolded tau could be a converged feature in FTLD-Tau pathology in different *MAPT* mutations.

We found that the progressive accumulation and extracellular release of misfolded tau and these phenotypes were inhibited by DREADDs in FTLD-Tau neurons, which was supported by previous reports showing that tau oligomers, an intermediate in the early stages of tau misfolding before overt fibrillization, cause increased intracellular calcium levels^24^,^25^ and neuronal death of healthy neurons^26^. Since DREADDs regulate calcium influx associated with neuronal activity^27^, intracellular calcium influx may contribute to tau misfolding, as reported in other neurodegenerative disease-associated pathogenic proteins^32^, and extracellular release of misfolded tau may accelerate inter-neuronal propagation of tau.

In this study, we detected severe cell death of FTLD-Tau neurons. We speculated that, compared to conventional differentiation method, transdifferentiation by introduction of transcription factor is likely a more stressful process that may enhance some cellular phenotypes, or that a lack of support from glia cells may have enhanced the vulnerability of FTLD-Tau neurons in the present model. We consider that neurons need to reach functional maturity in order for mutant tau to be toxic. Although the neurons in the present study were at a very early differentiation stage, these neurons exhibited glutamate receptor expression and functional property as shown by neurophysiological evaluation. Further investigation may be required to determine the precise neuronal function that allows mutant tau to be toxic.

These results suggested that FTLD-Tau patient iPSCs would be useful for investigating and understanding converging mechanism underlying tau mediated neurodegeneration due to different *MAPT* mutations, and a designer iPSC model with DREADDs to control neural activity is likely an approach that could be applied to other neurodegenerative diseases.

## Materials and Methods

### Ethics statement

Generation and use of human iPSC was approved by the Ethics Committee of the Department of Medicine and Graduate School of Medicine, Kyoto University, and Kawasaki Medical School. All methods were performed in accordance with the approved guidelines. Formal informed consent was obtained from all subjects.

### Generation of iPSCs and cell culture

Human iPSCs with a *MAPT* mutation were generated from fibroblasts or peripheral blood mononuclear cells (PBMCs) using episomal vectors for OCT3/4, Sox2, Klf4, L-Myc, Lin28, and dominant negative p53, as previously reported^33^, and were cultured on an SNL feeder layer with human iPSC medium (primate embryonic stem cell medium; ReproCELL, Yokohama, Japan) supplemented with 4 ng/ml basic FGF (Wako Chemicals, Osaka, Japan) and penicillin/streptomycin. As a control we used the 201B7 iPSC line^34^.

### Generation of isogenic human FTLD-Tau iPSC lines with CRISPR/Cas9 gene editing technology

For targeting *MAPT* by CRISPR/Cas9, we designed a guide RNA to target the 5′-GCAACGTCCAGTCCAAGTGTGG-3′ site using CRISPR Design (http://crispr.mit.edu/). The guide RNA oligonucleotide was inserted into the BamHI-EcoRI site in pHL-H1-ccdB plasmids to express the insert under the control of human H1 polymerase III promotor^35^. We inserted 5′ and 3′ homology arms with normal *MAPT* sequence, and puromycin-resistant cassette flanked with *piggyBac* terminal repeat into pBluescript SK(+) to construct the donor plasmid. For transfection of CRISPR/Cas9, one million iPSCs were electroporated with 10 μg of pHL-Hi guide RNA expression plasmids, 10 μg of pHL-EF1a-hcSpCas9 plasmids, and 10 μg of donor plasmids by NEPA21 electroporator (Nepagene, Chiba, Japan). At 4 days post-transfection, puromycin selection was performed for 10 days. The puromycin-resistant colonies were chosen and expanded for genomic DNA extraction and genotyping by PCR using the following primers: forward primer, 5′-AAATGCAGTCGTGGGAGACC-3′; reverse primer, 5′-GAGTCCCGAATCTCACGGAGACA-3′. All amplified PCR bands were further analyzed by Sanger sequencing to confirm that there were no sequence alterations at the homology arms. To remove the puromycin cassete, the transposase vector pHL-EF1α-hcPBase^36^ was electroporated into the targeted clones, and successful removal of the puromycin cassette was confirmed by PCR: forward primer, 5′-AAATGCAGTCGTGGGAGACC-3′; reverse primer, 5′-GAGTCCCGAATCTCACGGAGACA-3′. To exclude the possibility of re-integration of the puromycin-resistance cassette, we performed PCR of the puromycin cassette and confirmed its non-integration with the following primers: forward primer, 5′-CTGCTGCAACTTACCTCCGGGATG-3′; reverse primer, 5′-CCAATCCTCCCCCTTGCTGTCCTG-3′.

### Preparation of a *piggyBac* vector for expressing Ngn2 and introduction into iPSCs

To robustly generate homogenous neurons from iPSCs, we introduced the Ngn2 transcription factor into iPSCs using a *piggyBac* vector. This vector, containing *Ngn2* under control of the tetracycline operator rtTA and neomycin resistance gene, was generated from the KW110_PB_TA_ERN (Ef1a_rtTA_neo) vector backbone^37^. The generated vector was then co-transfected together with a pCyL43 vector encoding transposase into iPSCs using lipofectamin LTX (Thermo Fisher Scientific, Waltham, MA). After clone selection using neomycin, we established iPSCs carrying the tetracycline-inducible *Ngn2* construct.

### Generation of cortical neurons from iPSCs via induction of Ngn2

iPSCs were dissociated to single cells using acutase and plated on Matrigel-coated plastic plates or cover slips with the neural medium containing a 1:1 ratio of DMEM/F12 (Thermo Fisher Scientific) and Neurobasal (Thermo Fisher Scientific), 1% N2 supplement, 2% B27 supplement, 10 ng/ml brain-derived neurotrophic factor (BDNF, R&D Systems), 10 ng/ml glial cell-derived neurotrophic factor (GDNF, R&D Systems, Minneapolis, MN) and 10 ng/ml neurotrophin-3 (NT-3; R&D Systems) with 1 μg/ml doxycycline (TAKARA, Kusatsu, Japan).

### Introduction of DREADDs and analysis of misfolded tau after chemical stimulation for DREADDs

The designer receptor exclusively activated by designer drug (DREADD) technique was used to control neuronal activity. We introduced a human M4 muscarinic DREADD (M4D) into iPSCs using a tol2 vector^38^. A tol2 vector containing *M4D* under control of the CAG promoter and hygromycine resistance gene was generated from the pT2AL200R175-CAGGS-EGFP vector backbone following removal of the EGFP gene^38^. The generated vector was co-transfected along with a vector encoding transposase into the Ngn2-introduced iPSCs using lipofectamin LTX. After clone selection using hygromycin, we established iPSC lines carrying *M4D*. After the generated iPSCs were differentiated into neurons with doxycycline and neural medium for 8 days, the pharmacologically inert, designer drug clozapine-N-oxide (CNO) of 100 nM was added to culture medium to stimulate DREADD. On Day14, western blot analysis and dot blot analysis were performed.

### Reverse transcriptase PCR

Total RNA of cultured cells was extracted using an RNeasy Plus Mini kit (QIAGEN, Hilden, Germany). One microgram of RNA was reverse-transcribed using ReverTra Ace (TOYOBO, Osaka, Japan). PCR analysis was performed with TAKARA Ex Taq (TAKARA). Primer sets for 3 repeat tau and 4 repeat tau were used as previously reported; forward 5′-AAGTCGCCGTCTTCCGCCAAG-3′; reverse 5′-GTCCAGGGACCCAATCTTCGA-3′. RT-PCR products were evaluated on 3% agarose gel: RT-PCR products of 3 repeat tau and 4 repeat tau were 288 and 381 bp, respectively^17^.

### Quantitative RT-PCR

Total RNA of cultured cells on Day 7 was extracted using an RNeasy Plus Mini kit (QIAGEN). One microgram of RNA was reverse-transcribed using ReverTra Ace (TOYOBO). Quantitative PCR analysis was performed by reverse transcription reaction with SYBR Premix Ex TaqII (TAKARA) using Step One Plus (Applied Biosystems, Waltham, MA). Primer sets are listed in Table S3.

### Immunocytochemistry

Cells were fixed in 4% paraformaldehyde for 30 min at room temperature, washed with PBS, and permeabilized in PBS containing 0.2% Triton X-100 for 10 min at room temperature, followed by blocking for 30 min with Block Ace (Yukijirushi, Tokyo, Japan). After incubation with primary antibodies overnight at 4 °C, cells were washed three times with PBS and incubated with appropriate secondary antibodies for 1 h at room temperature. Cell images were acquired with Delta Vision (GE Healthcare, Chicago, IL) or IN Cell Analyzer 6000 (GE Healthcare). The numbers of cells were quantified with IN Cell Analyzer 6000 and IN CELL Developer toolbox software 1.9 (GE Healthcare). The following primary antibodies were used in this assay: βIII tubulin (1:2,000, Covance, Princeton, NJ), NeuN (1:500, Millipore, Darmstadt, Germany), TOC1 (1:1,000), Nanog (1:200, Abcam, Cambridge, UK), and SSEA4 (1:500, Millipore).

### Western blot analysis

Cells were harvested and dissolved in RIPA buffer containing 0.1% SDS, 150 mM NaCl, 1% NP-40, 0.5% deoxycholate, 50 mM Tris-HCl (pH8.0), protease inhibitor (Roche, Basel, Switzerland), and phosphatase inhibitor (Roche). After sonication, samples were centrifuged at 13,000 × g for 15 min at 4 °C. Protein concentrations in the supernatants were determined using the bicinchoninic acid (BCA) assay kit (Thermo Fisher Scientific). Total protein extracts (20 μg per lane) with or without 2-mercaptoethanol, reducing condition or non-reducing condition, respectively, were separated by size on 10% polyacrylamide gels and transferred to an Immobilon-P membrane (Millipore). Membranes were blocked with 5% skim milk, hybridized with appropriate antibodies, and visualized using the ECL Prime detection kit (GE Healthcare). Images were acquired on ImageQuant LAS 4000 (GE Healthcare). The following primary antibodies were used: MAP2 (1:1,000, CST, Danvers, MA), βIII tubulin (1:5,000, Covance), Tau12 (1:10,000, Millipore), TOC1 (1:5,000), and β-actin (1:5,000, SIGMA, St. Louis, MO).

It is noteworthy that a non-denaturing condition is required to assess the oligomeric form of tau using the TOC1 antibody, as this antibody has a linear epitope that is displayed in oligomers compared to monomers or filaments^23^. Using denaturing conditions does not allow for the distinction between these different tau species, oligomers, monomers, and/or filaments, using TOC1.

### Dot blot analysis

Cells were harvested and lysed in TBS containing protease inhibitor and phosphatase inhibitor. After sonication and centrifugation at 13,000 × g for 15 min, each of the lysate samples (1.2 μg/spot) was loaded on a nitrocellulose membrane (0.45 μm pore size, GE Healthcare). Cell culture medium was collected and centrifuged at 800 × g for 3 min to remove debris. 500 μl supernatant from each sample was concentrated to 50 μl using Vivaspin (GE Healthcare), with a molecular weight cut-off of the filtration membrane of 10 kD. 2 μl of each concentrated sample was loaded on a nitrocellulose membrane. Membranes were blocked with 5% skim milk, hybridized with appropriate antibodies, and visualized using Western Lightning Plus-ECL (PerkinElmer, Waltham, MA). Images were acquired on ImageQuant LAS 4000 (GE Healthcare). The following primary antibodies were used: Tau12 (1:10,000, Millipore) and TOC1 (1:5,000).

### Cell survival assay

Ngn2-introduced iPSCs were dissociated to single cells, and 5 × 10^4^ cells/well were plated on Matrigel-coated 96-well plates (BD Bioscience, Franklin Lakes, NJ) with neural medium containing 1 μg/ml doxycycline. Cells were fixed and stained on Days 8 and 21. The number of surviving neurons stained with NeuN was quantified by IN Cell Analyzer 6000 (GE Healthcare), and expressed as the number of neurons on Day 21/Day 8. To inhibit neuronal activation, D-2-amino-5-phosphonopentanoate (AP-5) (SIGMA) and 6-cyano-7-nitroquinoxaline-2, 3-dione (CNQX) (SIGMA) were added daily from Day 8 to Day 21, and the number of surviving neurons was evaluated on Day 21.

### Cell death assay

Ngn2-introduced iPSCs were dissociated to single cells, and 5 × 10^4^ cells/well were plated on Matrigel-coated 96-well plates (BD Bioscience) with neural medium containing 1 μg/ml doxycycline. Culture medium was changed on day 8, and propidium iodide (PI) staining was performed on day 10. Cells were incubated with 1.5μM PI (NACALAI TESQUE, Kyoto, Japan) for 30 min at 37 °C, and fixed in 4% paraformaldehyde. The number of PI stained cells was quantified by IN Cell Analyzer 6000.

### Enzyme-linked immunosorbent assay (ELISA) of misfolded tau

96-well plates (Greiner, Frickenhausen, Germany) were coated with 3 μg/ml TOC1 antibody in 0.05 M sodium carbonate buffer at 4 °C overnight. After washing and blocking with TBS-T containing 1% BSA, 100 μl of cultured medium was added, and incubation was carried out for 2 h at room temperature. Recombinant oligomeric tau protein was used to obtain a standard curve. For detection, the plates were incubated with 2 μg/ml affinity-purified rabbit polyclonal anti-human tau antibody, followed by sheep anti-rabbit IgG F(ab)’2 fragment linked to horseradish peroxidase (1:3,000; GE Healthcare). After incubation with tetramethylbenzidine solution (BD Bioscience) at room temperature for 30 min, absorbance at 450 nm was measured by VersaMax (Molecular Devices, Sunnyvale, CA). The anti-human tau antibody was raised against tau amino-terminus polypeptide (amino acids 19–33, according to the longest human tau isoform) by immunization of two rabbits.

### Calcium imaging assay

iPSCs were dissociated to single cells, and 5 × 10^4^ cells/well were plated on Matrigel-coated 96-well plates with neuronal medium containing 1 μg/ml doxycycline. On Day 10, electrically stimulated intracellular Ca^2+^ mobilization was measured using e-FDSS/μCELL (Hamamatsu Photonics, Hamamatsu, Japan)^39^. In brief, cells were incubated with 5 μM Fluo-8/AM (AAT Bioquest, Sunnyvale, CA) and 0.01% Pluronic F-127 (SIGMA) in neuronal medium for 30 min at 37 °C. After washing with PBS, the medium was changed to phenol red-free Neurobasal medium (Thermo Fisher Scientific). Electrical stimulations (Voltage: 20 V, Pulse width: 3 msec, Number: 50 times/stimulation, Frequency: 50 Hz) were delivered to each well of the 96-well plates, and fluorescence changes (excitation and emission wavelengths, 480 and 540 nm, respectively), expressed as a percentage of fluorescence recorded before and after stimulation, were recorded.

### Electrophysiological recordings

Whole-cell patch-clamp recordings were performed from iPSC-derived neurons. The recording micropipettes were filled with intracellular solution consisting of 140 mM KCl, 2 mM MgCl_2_, 10 mM HEPES, and 1 mM EGTA, adjusted to pH7.4 with NaOH. Cells were maintained at 30 °C during the experiment and were continuously superfused with oxygenated Krebs-Ringer solution consisting of 125 mM NaCl, 2.5 mM KCl, 1.25 mM NaH_2_PO_4_, 26 mM NaHCO_3_, 1 mM MgCl_2_, 2 mM CaCl_2_, and 20 mM glucose. Voltage-clamp and current-clamp recordings were made using an EPC9 amplifier (HEKA, Lambrecht, Germany), and data were analyzed with Patchmaster software (HEKA).

### Statistical analysis

Results were analyzed using one-way ANOVA followed by Tukey *post hoc* analysis or student’s *t*-test to determine statistical significance. Differences were considered significant at p < 0.05. Analyses were performed using SPSS software (IBM, Armonk, NY). All bar graphs represent mean ± SEM.

## Additional Information

**How to cite this article**: Imamura, K. *et al.* Calcium dysregulation contributes to neurodegeneration in FTLD patient iPSC-derived neurons. *Sci. Rep.* **6**, 34904; doi: 10.1038/srep34904 (2016).

## Supplementary Material

Supplementary Information
