# Supplementary material for: Calcium dysregulation contributes to neurodegeneration in FTLD patient iPSC-derived neurons
Source: Sci Rep. 2016 Oct 10;6:34904. doi: 10.1038/srep34904 (PMC5056519; doi:10.1038/srep34904)
Supplement: Supplementary Information [file srep34904-s1.pdf]

Supplementary Information

Title of manuscript:

Calcium dysregulation contributes to neurodegeneration in FTLD patient iPSC-derived neurons

Author list

Keiko Imamura, Naruhiko Sahara, Nicholas M. Kanaan, Kayoko Tsukita, Takayuki Kondo, Yumiko Kutoku, Yutaka Ohsawa, Yoshihide Sunada, Koichi Kawakami, Akitsu Hotta, Satoshi Yawata, Dai Watanabe, Masato Hasegawa, John Q. Trojanowski, Virginia M.-Y. Lee, Tetsuya Suhara, Makoto Higuchi, Haruhisa Inoue

Figure S1-S4

Figure legend of Figure S1-S4

Table S1-S3

Figure S1

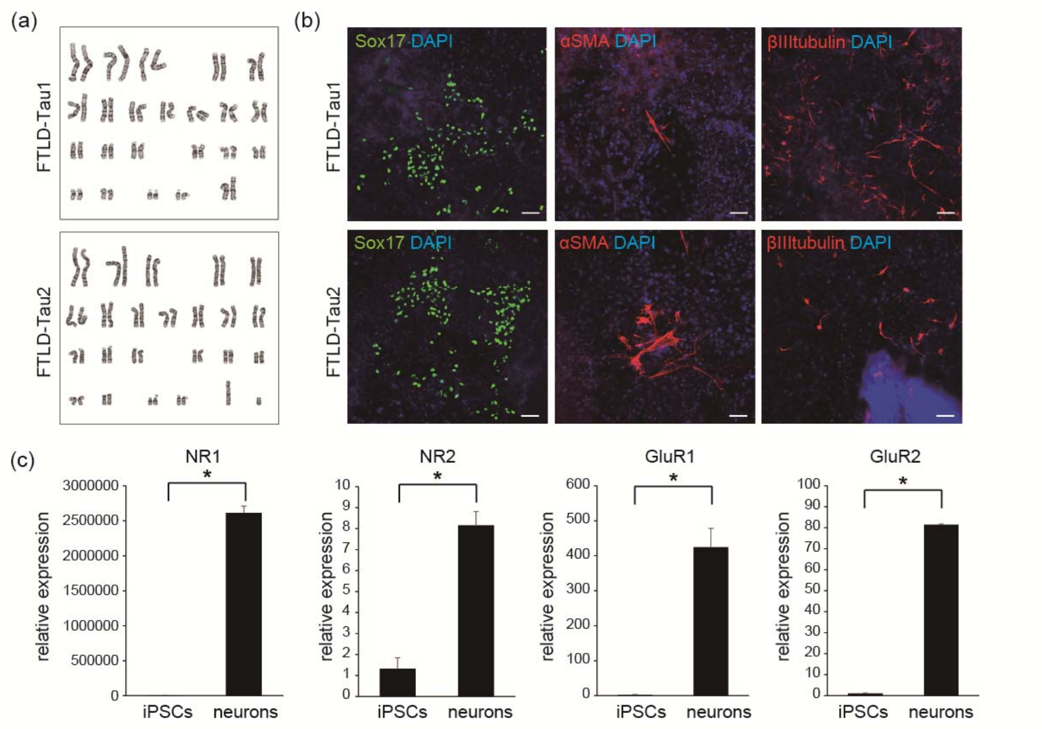

Figure S1. Characterization of iPSCs and generated neurons, related to Figure 1

(a) iPSCs presented normal karyotypes.

(b) Pluripotency of iPSCs was confirmed by in vitro three-germ layer assay. Scale bar = 50 μm.

(c) Generated neurons expressed mRNA of glutamate receptors such as NR1, NR2, GluR1, and GluR2 (n = 3; Student-*t* test, * $p < 0.05$).

Figure S2

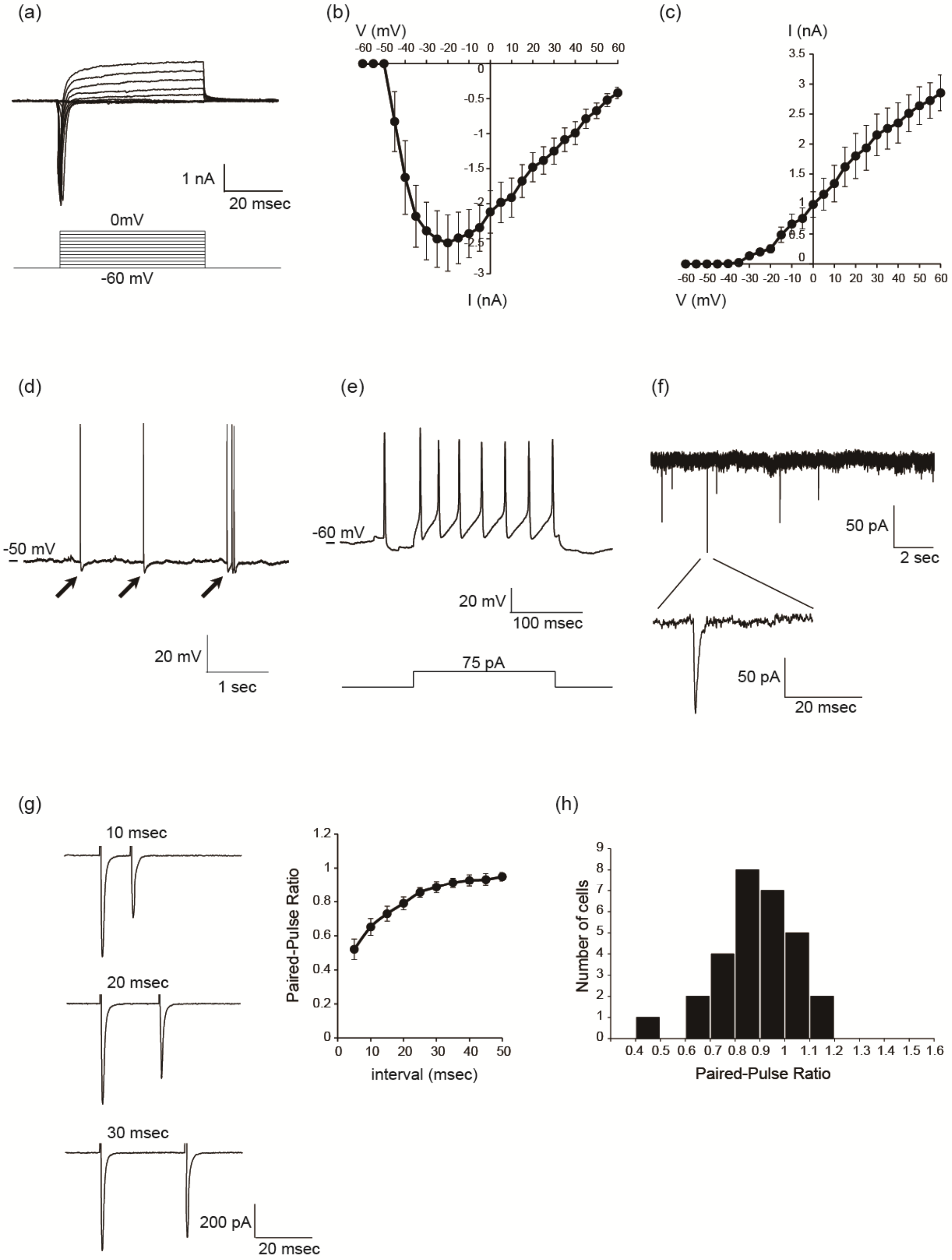

(a)
1 nA
20 msec
0mV
-60 mV
(b)
V (mV)
-60 -50 -40 -30 -20 -10 0 10 20 30 40 50 60
0
-0.5
-1
-1.5
-2
-2.5
-3
I (nA)
(c)
I (nA)
3.5
3
2.5
2
1.5
1
0.5
0
-60 -50 -40 -30 -20 -10 0 10 20 30 40 50 60
V (mV)
(d)
-50 mV
20 mV
1 sec
(e)
-60 mV
20 mV
100 msec
75 pA
(f)
50 pA
2 sec
50 pA
20 msec
(g)
10 msec
20 msec
30 msec
200 pA
20 msec
Paired-Pulse Ratio
1.2
1
0.8
0.6
0.4
0.2
0
0 10 20 30 40 50
interval (msec)
(h)
Number of cells
9
8
7
6
5
4
3
2
1
0
0.4 0.5 0.6 0.7 0.8 0.9 1 1.1 1.2 1.3 1.4 1.5 1.6
Paired-Pulse Ratio

Figure S2. Electrophysiological analysis of iPSC-derived neurons, related to Figure 1

To confirm whether iPSC-derived neurons were functional, the electrophysiological properties of control iPSC-derived neurons differentiated for 3 weeks were evaluated by patch-clamp analysis. Resting membrane potentials of iPSC-derived neurons were -52.7 ± 0.9 (mean ± SEM, n = 10).

(a) iPSC-derived neurons presented voltage-dependent Na^+ currents and K^+ currents. Representative traces of whole-cell currents measured in voltage-clamp mode were presented. Currents were evoked by 50 msec depolarizing steps from -60 mV to 0 mV in 5-mV increments from the holding potential of -60 mV.

(b) Current-voltage relationship of Na^+ currents in voltage-clamp. Cells were clamped at -60 mV and voltage was increased stepwise from -60 mV to +60 mV in 5-mV intervals. Peak amplitudes of inward whole-cell currents were measured (mean ± SEM, n = 10).

(c) Current-voltage relationship of K^+ currents in voltage-clamp. Cells were clamped at -60 mV and voltage was increased stepwise from -60 mV to +60 mV in 5-mV intervals. Amplitudes of outward currents were measured at the end of pulse (mean ± SEM, n = 10).

(d) Spontaneous action potentials were recorded in current-clamp mode from iPSC-derived neurons. Arrows show after hyperpolarization potentials.

(e) A representative trace of membrane potential in response to current injection. Membrane potential was recorded by injection of 75 pA depolarizing current from a resting membrane potential of -60 mV. According to the current injection, the train of action potentials was recorded.

(f) Representative continuous whole-cell recordings from iPSC-derived neuron holed at -60 mV. Spontaneous postsynaptic currents were recorded showing that iPSC-derived neurons had functional synapses.

To investigate whether iPSC-derived neurons exhibited synaptic plasticity, synaptic responses were evaluated following paired-pulse stimulation using differentiated neurons for 3-4 weeks.

(g) iPSC-derived neurons exhibited paired-pulse depression in response to paired-pulse stimulation. Whole cell recordings were made and synaptic response was evoked by 0.1mA of electrical stimulation of a synaptically connected cell. The representative averaged traces and the relationship of paired-pulse ratio and interstimulus interval were presented (n = 5 stimulations on the same cell). Paired-pulse depression of postsynaptic currents occurred at presynaptic paired-stimuli at intervals ranging from 5 msec to 50 msec. Paired-pulse ratio: second postsynaptic potential / first postsynaptic potential.

(h) The histogram summarizes data of paired-pulse stimulation from 29 cells and shows the distribution of paired-pulse ratios. 75.9% of iPSC-derived neurons presented paired-pulse depression.

Figure S3

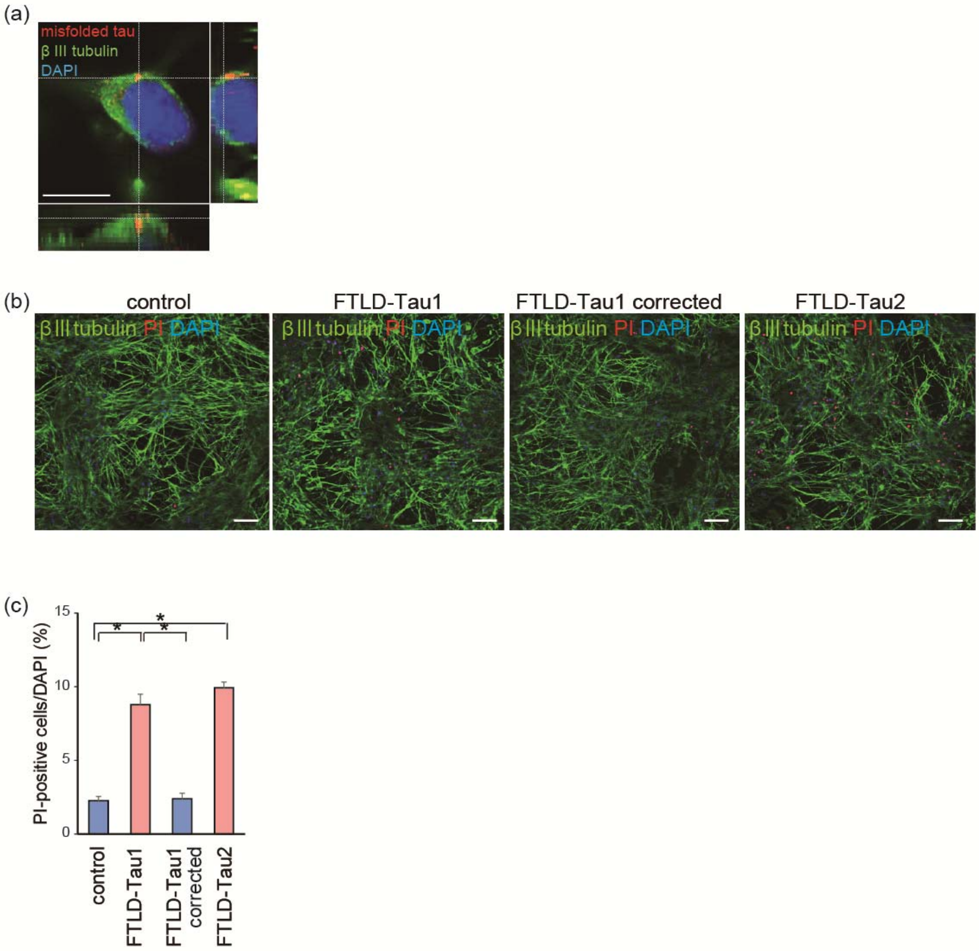

Figure S3. Evaluation of misfolded tau and neuronal death, related to Figures 2 and 3

(a) Orthogonal analysis of oligomeric tau in FTLD-Tau2 neurons with TOC1 antibody. Scale bar = 10 μm.

(b,c) Evaluation of neuronal death using PI staining. PI-positive cells were increased in FTLD-Tau1 and Tau-2 compared with control. Scale bar = 50 μm (n = 6; one-way ANOVA, $p < 0.05$; *post hoc* test, * $p < 0.05$).

Figure S4

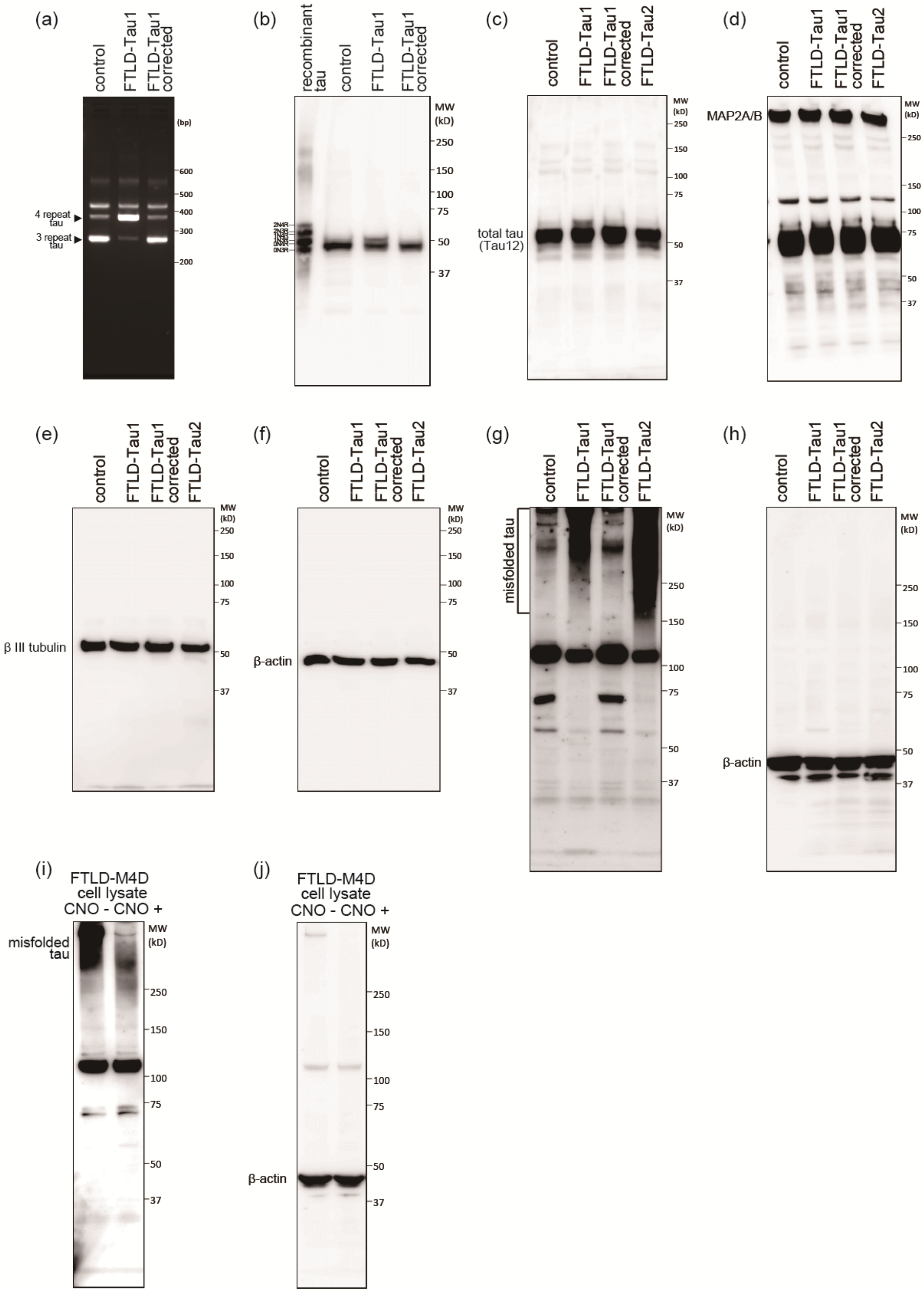

(a)
control
FTLD-Tau1
FTLD-Tau1 corrected
(bp)
600
500
400
300
200
4 repeat tau
3 repeat tau
(b)
recombinant tau
control
FTLD-Tau1
FTLD-Tau1 corrected
MW (kD)
250
150
100
75
50
37
(c)
control
FTLD-Tau1
FTLD-Tau1 corrected
FTLD-Tau2
MW (kD)
total tau (Tau12)
(d)
control
FTLD-Tau1
FTLD-Tau1 corrected
FTLD-Tau2
MAP2A/B
MW (kD)
(e)
control
FTLD-Tau1
FTLD-Tau1 corrected
FTLD-Tau2
β III tubulin
MW (kD)
(f)
control
FTLD-Tau1
FTLD-Tau1 corrected
FTLD-Tau2
β-actin
MW (kD)
(g)
control
FTLD-Tau1
FTLD-Tau1 corrected
FTLD-Tau2
misfolded tau
MW (kD)
(h)
control
FTLD-Tau1
FTLD-Tau1 corrected
FTLD-Tau2
β-actin
MW (kD)
(i)
FTLD-M4D cell lysate
CNO - CNO +
misfolded tau
MW (kD)
(j)
FTLD-M4D cell lysate
CNO - CNO +
β-actin
MW (kD)

Figure S4. Full-length pictures of gel and blots presented in the main figures, related to Figure 1, 2, and 4.

(a) Full-length picture of the gel presented in Figure 1(e).

(b) Full-length picture of the blot presented in Figure 1(f).

(c-f) Full-length pictures of the blots presented in Figure 2(b).

(g,h) Full-length pictures of the blots presented in Figure 2(f).

(i,j) Full-length pictures of the blots presented in Figure 4(b).

Table S1. iPSC lines

	control	FTLD-Tau1	FTLD-Tau1 corrected	FTLD-Tau2
**clone name at establishment**	201B7	FTD2E1	FTD2E1 corrected 36-20	ND40071E11
**gender**	female	female	female	male
**onset age**	N.A.	39	N.A.	56
**age at diagnosis**	N.A.	40	N.A.	63
**biopsy age**	36	52	52	67
**genotype**	N.A.	*MAPT* (intron 10+14C→T)	N.A.	*MAPT* (R406W)
**origin**	fibroblast	peripheral blood mononuclear cell		fibroblast*
**reprogramming**	retrovirus	episomal	episomal	episomal

*: obtained from Coriell Institute.

Table S2. tau antibodies used in the manuscript

antibody	target	host species	supplier
**Tau12**	human tau	Ms (IgG)	Millipore
**TOC1**	oligomeric tau	Ms (IgM)	Kanaan/Binder Lab

Table S3. Primer list for qPCR

primer list	sequences (5' to 3')
NR1_F	GTCCAAGGCAGAGAAGGTGC
NR1_R	CTCGCTGGCAGAAAGGATGA
NR2_F	GGGTGAGCGCTGAGAATCG
NR2_R	GCAGCAGGGCTCGCAG
GluR1_F	GGGTCTGCCCTGAGAAATCC
GluR1_R	TCAGAGCGCTTGTCTTGTCC
GluR2_F	AAACTCAGTGAGCAAGGCGT
GluR2_R	GGGCACTGGTCTTTTCCTTACT
GAPDH_F	TCCACTGGCGTCTTCACC
GAPDH_R	GGCAGAGATGATGACCCTTTT
